# The Anti-Inflammatory Effect of SDF-1 Derived Peptide on *Porphyromonas gingivalis* Infection via Regulation of NLRP3 and AIM2 Inflammasome

**DOI:** 10.3390/pathogens13060474

**Published:** 2024-06-04

**Authors:** Si Yeong Kim, Min Kee Son, Jung Hwa Park, Hee Sam Na, Jin Chung

**Affiliations:** 1 Department of Oral Microbiology, School of Dentistry, Pusan National University, Yangsan 50612, Republic of Korea; gji0307@naver.com (S.Y.K.); blackbody23@hanmail.net (M.K.S.); pajuha2518@pusan.ac.kr (J.H.P.); heesamy@pusan.ac.kr (H.S.N.); 2 Oral Genomics Research Center, Pusan National University, Yangsan 50612, Republic of Korea; 3 BK21 PLUS Project, Dental Research Institute, School of Dentistry, Pusan National University, Yangsan 50612, Republic of Korea

**Keywords:** periodontitis, inflammation, NLRP3, AIM2, ASC

## Abstract

(1) **Background**: Peptides are appealing as pharmacological materials because they are easily produced, safe, and tolerable. Despite increasing gum-care awareness, periodontitis is still prevalent and is influenced by factors like high sugar consumption, smoking, and aging. *Porphyromonas gingivalis* is considered a major etiologic agent of periodontitis and activates the NLR family pyrin domain containing 3 (NLRP3) but is absent in melanoma 2 (AIM2) inflammasomes, resulting in pro-inflammatory cytokine release. (2) **Methods**: We examined the anti-inflammatory effects of 18 peptides derived from human stromal cell-derived factor-1 (SDF-1) on THP-1 macrophages. Inflammation was induced by *P. gingivalis*, and the anti-inflammatory effects were analyzed using molecular biological techniques. In a mouse periodontitis model, alveolar bone resorption was assessed using micro-CT. (3) **Results**: Of the 18 SDF-1-derived peptides, S10 notably reduced IL-1β and TNF-α secretion. S10 also diminished the *P. gingivalis*-induced expression of NLRP3, AIM2, ASC (apoptosis-associated speck-like protein), caspase-1, and IL-1β. Furthermore, S10 attenuated the enhanced TLR (toll-like receptor) signaling pathway and decreased the phosphorylation of nuclear factor-κB (NF-κB) and mitogen-activated protein kinases (MAPKs). In addition, S10 mitigated alveolar bone loss in our *P. gingivalis*-induced mouse model of periodontitis. (4) **Conclusions**: S10 suppressed TLR/NF-κB/NLRP3 inflammasome signaling and the AIM2 inflammasome in our *P. gingivalis*-induced murine periodontitis model, which suggests that it has potential use as a therapeutic treatment for periodontitis.

## 1. Introduction

Periodontitis is an inflammatory infection caused by oral pathogens, which causes the ongoing destruction of tooth-supporting tissues, the formation of periodontal pockets, and, ultimately, tooth loss [1,2]. The Gram-negative anaerobic bacterium *Porphyromonas gingivalis (P. gingivalis)* is widely acknowledged to be a major causative pathogen of periodontitis [3]. *P. gingivalis* acts by disrupting immune homeostasis in the host. Its virulence factors, which include lipopolysaccharide (LPS), proteases, and fimbriae, promote dental biofilm formation and adversely influence various components of the host’s immune system, thus subverting the immune response or inducing an inflammatory environment [4].

Patients with severe periodontitis exhibit significant increases in the expression of interleukin-1β (IL-1β), tumor necrosis factor-α (TNF-α), nod-like receptor protein 3 (NLRP3), and their absence in melanoma 2 (AIM2) inflammasomes in the gingival tissues [5,6]. NLRP3 expression is elevated in the serum and saliva of periodontitis patients, and AIM2 expression has been confirmed in the gingival tissues of both periodontitis and gingivitis patients [7,8,9]. The NLRP3 inflammasome plays a crucial role in the activation of the cysteine protease caspase-1 in response to microbial and non-microbial stimuli, and this activation leads to the secretions of inflammatory cytokines, such as IL-1β, and contributes to host defense against infectious insults [10]. Mechanistically, *P. gingivalis* increases the secretion of IL-1β and activates the NLRP3 inflammasome in human immune cells [11,12,13]. Furthermore, immune response-induced TNF-α contributes to the regulation of the transcription levels of NLRP3 inflammasome components [14]. Conversely, the AIM2 inflammasome is a “canonical” inflammasome that serves a dual role as an inflammasome that regulates innate immune responses and as a cytosolic DNA detector [15]. Furthermore, transfections of bacterial or viral DNA lead to AIM2 inflammasome activation and IL-1β secretion [16].

Peptide therapy is potent and efficient and causes minimal immune response. This type of therapy is highly specific, with low accumulation and drug interaction characteristics. In addition, peptides are less complex to produce and, thus, are cheaper than protein-based drugs, which makes them valuable for drug development and therapeutic applications [17]. Peptide therapy is also being employed in the treatment of periodontal diseases. Thousands of anti-bacterial peptides have been discovered and, in the field of periodontics, more than 45 anti-microbial peptides have been isolated or synthesized [18]. For example, LL-37 has anti-microbial activity against *Aggregatibacter actinomycetemcomitans* and *Prevotella intermedia*, while lactoferrin has anti-microbial activity against *P. gingivalis* and *P. intermedia* [18]. Also, MHP1 and [K6T] P8 are peptide therapeutics that regulate inflammatory responses in conditions such as ischemic stroke and rheumatoid arthritis [19]. While there is a considerable volume of literature on anti-inflammatory peptide drugs, comparatively little research has been conducted on antimicrobial peptide drugs for treating periodontitis [20,21]. However, the use of antimicrobial peptides often poses the problem of increasing resistance and dysbiosis among bacteria. In other words, the excessive use of antimicrobial peptides can induce bacterial resistance and disrupt the healthy balance of microorganisms. This underscores the need for further research on anti-inflammatory peptide therapies.

Stromal cell-derived factor (SDF)-1 is a critical inflammatory CXC chemokine that plays a key role in the homing of bone marrow and stromal cells to tissues [22]. Furthermore, it has been demonstrated that the expression of SDF-1 is elevated in various models of inflammation, such as in allergic airway disease and rheumatoid arthritis models, and it also transpires that SDF-1 plays a role in regulating diverse cellular homeostatic mechanisms [23,24]. In addition, it was recently proposed in a rheumatoid arthritis model study that SDF-1 is a potential target for NLRP3-dependent mechanisms [25].

In this study, we investigated whether SDF-1-derived peptides influence the activation of NLRP3 and AIM2 inflammasomes in THP-1 macrophages infected with *P. gingivalis*. In addition, we confirmed their effectiveness in a mouse model of *P. gingivalis*-induced periodontitis.

## 2. Materials and Methods

### 2.1. SDF-1-Derived Peptides

The SDF-1-derived peptides and their nucleotide sequences were donated by Dr. Hyung-geun Kim of Chonnam National University. The sequences were designed to encompass all known human SDF-1 isoforms, based on the SDF-1 isoform delta precursor sequence (National Center for Biotechnology Information database; NP_001171605.1). To further investigate SDF-1 derived peptide 10 (S10), the peptide was re-synthesized, based on the sequence provided by PeproTech (PeproTech, Cranbury, NJ, USA), resulting in a total of 18 peptides, each divided into 10 amino acids.

### 2.2. Bacterial Culture

*P. gingivalis* (strain 33277, Korean Collection Type Cultures, Daejeon, Republic of Korea) was grown under anaerobic conditions at 37 °C in Gifu anaerobic medium (GAM) (Nissui Seiyaku, Tokyo, Japan) supplemented with 5 μg/mL of hemin (Sigma Aldrich, St. Louis, MO, USA) and 0.5 mg/mL of vitamin K_1_ (Sigma Aldrich, St. Louis, MO, USA) to an optical density of 1.0 (at 650 nm), which was estimated to contain 10^9^ colony-forming units (CFU)/mL. Bacteria were washed and resuspended in Roswell Park Memorial Institute (RPMI) medium (WelGENE, Daegu, Republic of Korea) before being used to infect THP-1-derived macrophages at an MOI (multiplicity of infection) of 100.

### 2.3. Cell Culture

THP-1 cells (Korean Cell Line Bank, Seoul, Republic of Korea) were cultured in RPMI 1640 medium (WelGENE, Daegu, Republic of Korea) supplemented with 10% heat-inactivated fetal bovine serum (FBS; WelGENE, Daegu, Republic of Korea). The cells were differentiated into macrophages by treating them with 50 μg/mL of Phorbol 12-myristate 13-acetate (PMA; Sigma Aldrich, St. Louis, MO, USA) for 24 h. The macrophages were pretreated with SDF-1-derived peptides (1 μg/mL) for 30 min and then infected with *P. gingivalis* for 18 h.

### 2.4. Cytokine Analysis

After peptide pretreatment and *P. gingivalis* infection, the culture media were analyzed using an ELISA (enzyme-linked immunosorbent assay) kit (Biolegend, San Diego, CA, USA) to determine the cytokine (IL-1β and TNF-α) levels. Absorbances were read using a microplate reader (Allsheng, Hangzhou, China). In this study, *n* = 3 indicates that each experimental condition was tested three times independently, to ensure reliability and reproducibility.

### 2.5. Western Blotting

The cells (THP-1-derived macrophages) were lysed in radioimmunoprecipitation (RIPA) buffer (Cell Signaling, Beverly, MA, USA) supplemented with complete EDTA (ethylene-diamine-tetra-acetic acid)-free protease inhibitor (Sigma Aldrich, St. Louis, MO, USA) and a phosphatase inhibitor cocktail (Genedepot, Katy, TX, USA). Cell lysates, containing equal amounts of protein (20 μg), were separated by SDS-PAGE (sodium dodecyl sulfate-polyacrylamide gel electrophoresis) and transferred onto PVDF (polyvinylidene difluoride) membranes (Merck Millipore, Burlington, MA, USA), which were then immunoblotted using the following specific antibodies: NLRP3 (Adipogen, San Diego, CA, USA), AIM2 (Abcam, Cambridge, UK), ASC (Adipogen, San Diego, CA, USA), caspase-1 (Novus, Littleton, CO, USA), IL-1β (Santa Cruz, CA, USA), TLR2 (Invitrogen, Carlsbad, CA, USA), TLR4 (Santa Cruz, CA, USA), MYD88 (Santa Cruz, CA, USA), TRIF (Santa Cruz, CA, USA), TRAF6 (Santa Cruz, CA, USA), phospho-NF-κB (Santa Cruz, CA, USA), NF-κB (Cell Signaling, Beverly, MA, USA), phospho-JNK (Cell Signaling, Beverly, MA, USA), JNK (Cell Signaling, Beverly, MA, USA), phospho-p38 (Cell Signaling, Beverly, MA, USA), p38 (Cell Signaling, Beverly, MA, USA), phospho-ERK (Cell Signaling, Beverly, MA, USA), ERK (Cell Signaling, Beverly, MA, USA) and β-actin (Santa Cruz, CA, USA). Signal detection was performed using enhanced chemiluminescence, and the resulting signals were visualized using the Super Signal West Femto maximum sensitivity substrate (Pierce, Rockford, IL, USA) and a LAS-4000 Immuno-Image Analyzer (Fuji Film, Tokyo, Japan). Band intensities were quantified using Image J software (version 1.53t, Fujifilm, Tokyo, Japan) with normalized versus β-actin. In this study, *n = 3* indicates that each experimental condition was tested three times independently to ensure reliability and reproducibility.

### 2.6. Real-Time PCR

The total RNA was extracted using the RNeasy Mini kit (Qiagen, Valencia, CA, USA), and cDNA was synthesized with a reverse transcription system (Bioneer, Daejeon, Republic of Korea). The cDNA was subjected to RT-PCR (real-time polymerase chain reaction) using SYBR Green PCR Master Mix (Applied Biosystems, Foster City, CA, USA) and an ABI 7500 real-time PCR system (Qiagen, Valencia, CA, USA). The primer sequences were as follows: human GAPDH, 5′-ACAACTTTGGTATCGTGGAAGG’ (forward) and 5′-GCCATCACGCCACAGTTTC-3′ (reverse); human NLRP3, 5′-CCACAAGATCGTGAGAAAACCC-3′ (forward) and 5′-CGGTCCTATGTGCTCGTCA-3′ (reverse); human AIM2, 5′-AGCAAGATATTATCGGCACAGTG-3′ (forward) and 5′-GTTCAGCGGGACATTAACCTT-3′ (reverse); human ASC, 5′-TGGATGCTCTGTACGGGAAG-3′ (forward) and 5′-CCAGGCTGGTGTGAAACTGAA-3′ (reverse); human Caspase-1, 5′-TTTCCGCAAGGTTCGATTTTCA-3′ (forward) and 5′-GGCATCTGCGCTCTACCATC-3′ (reverse); human IL-1β, 5′-TTCGACACATGGGATAACGAGG-3′ (forward) and 5′-TTTTTGCTGTGAGTCCCGGAG-3′ (reverse). In this study, *n* = 3 indicates that each experimental condition was tested three times independently to ensure reliability and reproducibility.

### 2.7. ASC Speck Quantification

ASC-GFP-THP-1 cells, prepared as described previously [12], were seeded in 8-well chambers and primed with PMA (50 ng/mL), pretreated with S10 (1 μg/mL) for 30 min, and then infected with *P. gingivalis* at an MOI of 100 for 18 h. ASC-GFP speck-containing cells (at least 300) were counted using a confocal laser-scanning microscope (LSM 700, Carl Zeiss, Prenzlauer, Berlin, Germany). Percentages of the total cells containing ASC specks were then calculated.

### 2.8. Mouse Periodontitis Model

The number of animals per group was determined based on the study design’s requirements for achieving statistically significant results. In each group, the number of animals used was as follows: control group = 4, *P. gingivalis* (Pg) group = 6, Pg + S10 group = 6, and S10 group = 4. The study sample size was determined based on the findings from our previous research [26,27]. To ensure the reliability of the experimental results, the animals were randomly assigned to groups using a random number generation method. Six-week-old C57BL/6 mice were infected with *P. gingivalis* to induce experimental periodontitis. Initially, the mice were administered sulfamethoxazole-trimethoprim ad libitum in their drinking water for 3 days to depress the commensal microbiota, which has the potential to interfere with *P. gingivalis* infection. and then rested for 3 days. On day 6, the mice were inoculated with *P. gingivalis* and randomly divided into 4 groups, namely, an untreated normal control group, a *P. gingivalis*-infected control group (1 × 10^8^ CFU/kg body weight; the *P. gingivalis* group), an S10-treated periodontitis group (1 mg/kg body weight; the *P. gingivalis* + S10 group), and an S10-treated group (S10 group). In the *P. gingivalis* and *P. gingivalis* + S10 groups, *P. gingivalis* (1 × 10^8^ CFU/kg body weight) suspended in 100 µL of 2% carboxymethyl cellulose was administered by oral gavage using a Zonde needle every 2 days for 2 weeks. In the *P. gingivalis* + S10 and S10 groups, S10 (1 mg/kg body weight) suspended in 100 µL of 2% carboxymethyl cellulose was administrated by oral gavage to the mice using a Zonde needle 30 min before the *P. gingivalis* injection for 2 weeks, and, subsequently, every 2 days for an additional 2 weeks. These S10 dose schedules were based on previous reports [26,27]. The mice were euthanized and evaluated on day 49. The study protocol was approved by the Institutional Animal Care and Use Committee of Pusan National University (PNU-2024-0273).

### 2.9. Micro-CT Scanning and Analysis of Alveolar Bone Loss

Mandibular bone specimens were prepared to assess alveolar bone resorption. Micro-CT scanning was conducted using the InspeXio SMX-90CT system (Shimadzu Science, Tokyo, Japan) using the following settings: 90 kV, 110 µA, and a 0.5 mm aluminum attenuation filter. Scans were reconstructed to produce three-dimensional models. Regions of interest (ROI) were defined as cuboidal bone bodies encompassing the roots and covering the entire range from the most mesial to the most distal aspects of the lower three molar roots. Standardized anatomical reference positioning was achieved using the TRI/3D Bone software (version 7.0, Ratoc, Tokyo, Japan). The extent of alveolar bone loss was calculated by summing the areas between the cementoenamel junctions (CEJ) and alveolar bone crests (ABCs) of teeth using ImageJ software (version 1.53t, Fujifilm, Tokyo, Japan). All measurements were conducted by a single-blinded examiner to ensure consistency and impartiality.

### 2.10. Statistics

All data are presented as means ± the standard deviation (SD). GraphPad Prism 9.5 software (GraphPad, San Diego, CA, USA) was used for statistical analysis. The normality of the variables was checked via the Shapiro–Wilk test, then Student’s *t*-test was performed for normally distributed samples or the Mann–Whitney test for nonparametric samples. The comparisons among multiple groups were analyzed by a one-way analysis of variance (ANOVA) with a Dunnett post hoc test. Statistical significance was considered at a *p*-value < 0.05, indicating significant differences between groups.

## 3. Results

### 3.1. SDF-1-Derived Peptides, Especially S10, Inhibited the Secretion of IL-1β and TNF-α by P. gingivalis-Infected THP-1 Macrophages

**Figure 1:**
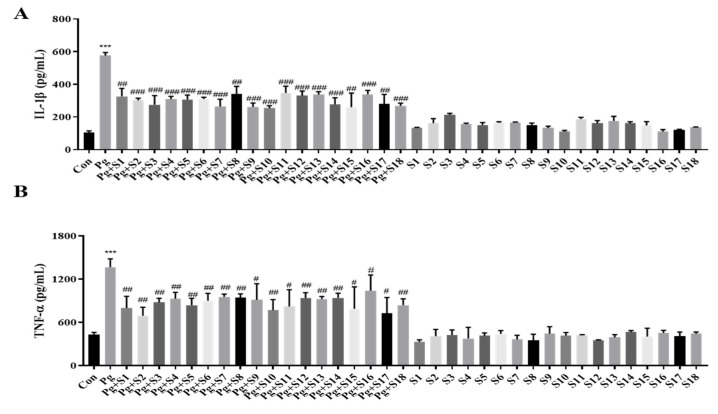
SDF-1-derived peptides suppressed the secretion of IL-1β and TNF-α. (**A**,**B**) THP-1 macrophages were pretreated with each SDF-1 derived peptide (1 μg/mL) for 30 min and were then infected with *P. gingivalis* (MOI 100). IL-1β (**A**) and TNF-α (**B**) secretion levels were measured by ELISA (*n =* 3). *** *p*-value < 0.001 versus untreated group (Con); ^#^ *p*-value < 0.05; ^##^ *p*-value < 0.01; ^###^ *p*-value < 0.001 versus *P. gingivalis* infection (Pg).

**Figure 2:**
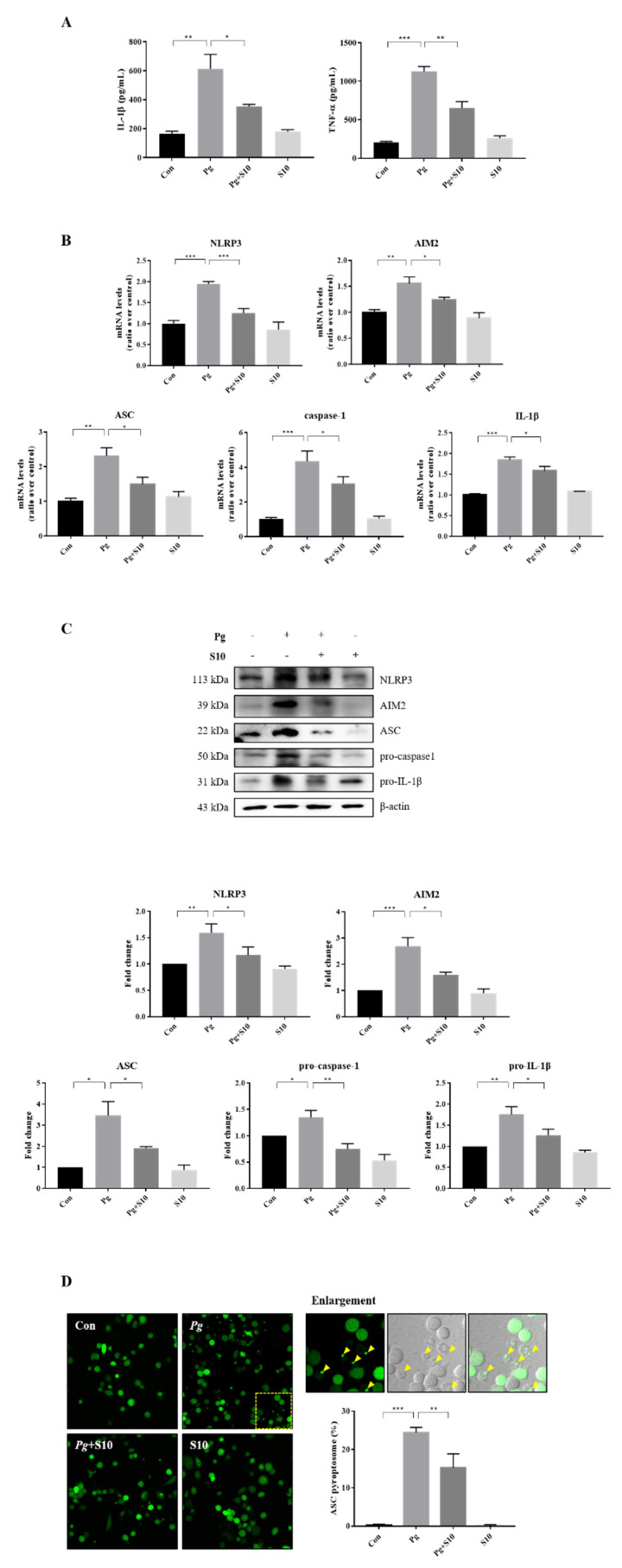
S10 inhibited the activation of NLRP3 and AIM2 inflammasomes by *P. gingivalis*. (**A**) THP-1 macrophages were pretreated with S10 (1 μg/mL) for 30 min and were then infected with *P. gingivalis* (MOI 100) for 18 h. The secreted levels of IL-1β and TNF-α in the supernatant were determined by an ELISA (*n =* 3). (**B**) Real-time PCR was conducted to quantify its effects on NLRP3 and AIM2 inflammasome components; the results are presented in the graph (*n =* 3). (**C**) Cell lysates were subjected to Western blot analysis, and representative immunoblots and graphs of protein levels versus β-actin for each protein are shown (*n =* 3). Blot densities are expressed relative to treatment-naïve controls. (**D**) PMA-primed ASC-GFP-THP-1 cells were pretreated with S10 for 30 min and then infected with *P. gingivalis* for 18 h. “Pg” in the images indicates the ASC speck, indicated by yellow arrows (original magnification 200×). The graph shows the percentage of total cells containing ASC. * *p*-value < 0.05; ** *p*-value < 0.01; *** *p*-value < 0.001.

We designed 18 small peptides complementing all isoforms of human SDF-1 and labeled them S1 to S18. Each of these peptides consisted of 15 amino acids, and the last 5 amino acids at the C-terminal were included in the sequence of the next peptide in the sequence. Based on the MTT results, we aimed to use the highest concentration that is non-toxic to the cells, and, consequently, decided to use a concentration of 1 µg/mL. An ELISA was performed on the pro-inflammatory cytokines, IL-1β (Figure 1A) and TNF-α (Figure 1B), to screen the anti-inflammatory effects of peptides S1 to S18. THP-1 macrophages treated with *P. gingivalis* displayed a 5-fold increase in IL-1β secretion and a 2.5-fold increase in TNF-α secretion compared to treatment-naïve controls. All 18 SDF-1-derived peptides significantly suppressed these cytokine elevations. In particular, S10 effectively reduced the *P. gingivalis*-induced secretion of IL-1β and TNF-α cytokines while not causing significant changes during S10 single treatment (Figure 2A).

### 3.2. The Activation of the NLRP3 and AIM2 Inflammasomes Was Attenuated by Pretreatment with S10

The expression levels of the NLRP3 and AIM2 inflammasome components were investigated to probe the mechanism responsible for pro-inflammatory cytokine inhibition by S10. THP-1 macrophages infected with *P. gingivalis* exhibited significant increases in the gene expression of NLRP3, AIM2, ASC, Caspase-1, and IL-1β versus treatment-naïve controls. Interestingly, S10 significantly reduced this gene expression (Figure 2B). Furthermore, these results mirrored the protein expression levels (Figure 2C). Inflammasome assembly is initiated by the activation of sensor proteins and leads to the recruitment and oligomerization of the ASC adapter protein. Oligomerized ASC then forms a structure known as a ‘speck’, which recruits pro-caspase-1 [28,29,30]. Thus, we counted the ASC puncta in ASC-GFP-THP-1 cells to investigate the effect of S10 on ASC speck formation. The numbers of ASC puncta increased after *P. gingivalis* infection, and these increases were significantly diminished by S10 (Figure 2D). These observations support the notion that S10 inhibits NLRP3 and AIM2 inflammasome formation and the speck-associated inflammatory cell death mechanism, and reduces the release of pro-inflammatory cytokines from *P. gingivalis*-infected cells.

### 3.3. The Suppression of NLRP3 and AIM2 Inflammasomes by S10 Was Related to the NF-kB and TLR2/4 Signaling Pathways

**Figure 3:**
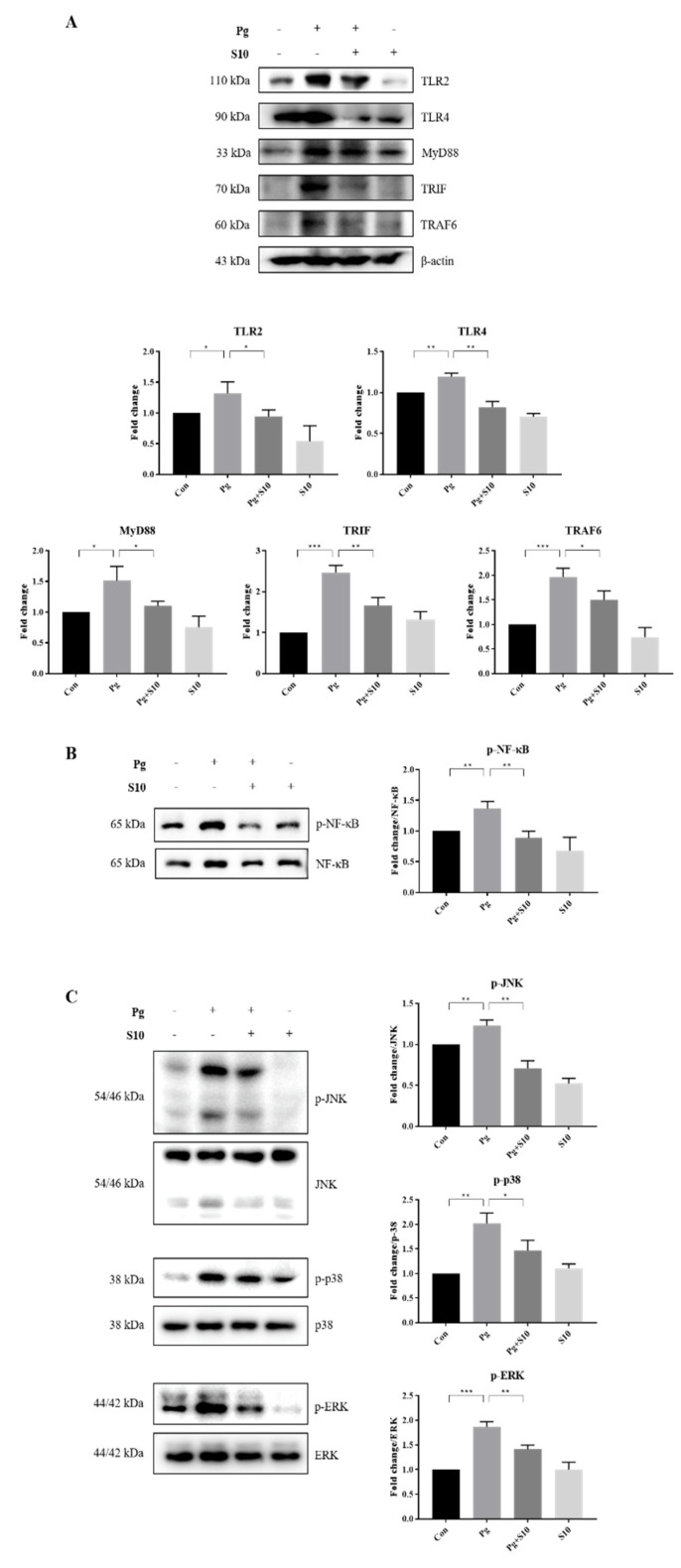
S10 suppressed the *P. gingivalis*-induced activation of the TLR2/4 and NF-κB/MAPK signaling pathways. (**A**) Cell lysates of THP-1-derived macrophages, pretreated with S10 (1 μg/mL) for 30 min and infected with *P. gingivalis* (MOI 100) for 18 h, were studied via Western blot analysis. TLR2/4 and their downstream signaling proteins were analyzed, and representative figures, along with densitometric graphs normalized versus β-actin, are shown (*n =* 3). (**B**,**C**) The phosphorylation levels of NF-Κb and MAPK were evaluated using dedicated antibodies targeting each phosphorylated site, and normalization was conducted versus total protein levels (*n =* 3). * *p*-value < 0.05; ** *p*-value < 0.01; *** *p*-value < 0.001.

The NLRP3 inflammasome is primed by microbial stimuli through the activation of TLRs and the NF-κB/MAPK signaling pathway, and this leads to the increased transcription of genes associated with inflammatory responses, such as NLRP3 and pro-IL-1β [31]. The protein expressions of key components involved in the TLR signaling pathway, including TLR2, TLR4, MyD88, TRAF6, and TRIF, were examined to investigate the mechanism responsible for inflammasome activation by *P. gingivalis*. Notably, S10 significantly decreased *P. gingivalis*-induced increases in the TLR pathway components (Figure 3A).

LPS-induced TLR signals are transmitted through NF-KB and MAPKs, including p38, ERK1/2, and JNK [32]. We observed that S10 suppressed the TLR signaling-induced phosphorylation levels of NF-κB and MAPKs (Figure 3B,C), indicating that S10 inhibits the NLRP3 inflammasome by mediating the TLR2/4 signaling pathway.

### 3.4. S10 Inhibited the P. gingivalis-Induced Loss of the Mandibular Alveolar Bone in Mice

**Figure 4:**
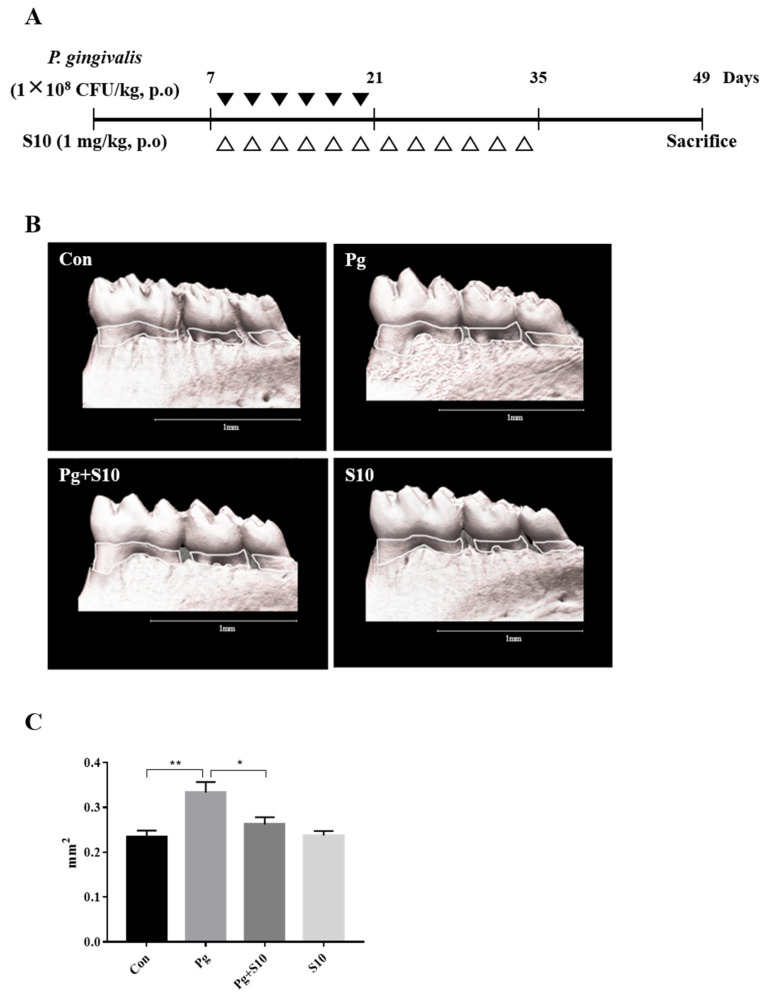
S10 alleviated the alveolar bone loss caused by *P. gingivalis* infection. (**A**) Details of the S10 treatment protocol. (**B**,**C**) Micro-CT images were used to measure alveolar bone areas after orally administering *P. gingivalis* with or without S10 treatment. Areas between the cementoenamel junctions and alveolar bone crests of the three molars in the captured images were measured using ImageJ. Images are representative of the three groups, and graphs summarize the measured alveolar bone areas (*n =* 6). * *p*-value < 0.05; ** *p*-value < 0.01.

A mouse periodontitis model was designed to determine whether the anti-inflammatory effects of S10 that were observed in vitro were evident in vivo. S10 was administered as shown in Figure 4A. Micro CT scans showed that the *P. gingivalis* infection resulted in significant alveolar bone loss compared to normal controls and that S10 mitigated this effect (Figure 4C).

## 4. Discussion

Periodontitis is an inflammatory disease, often associated with the presence of *P. gingivalis* in subgingival biofilms, which destroys tissues by releasing pro-inflammatory cytokines such as IL-1β and TNF-α [33]. Combinatorial mechanical and chemical treatments, including antibiotics and NSAIDs, provide a means of treating periodontitis, but the heterogeneous nature of periodontal diseases limits these treatments; for example, NSAIDs can lead to adverse effects like gastrointestinal problems [34]. To address these shortcomings, researchers are exploring whether peptide drugs offer alternatives to antibiotics and NSAIDs and the means of treating various conditions [35,36,37]. This approach offers the advantages of high specificity and biological activity, metabolic stability, versatility across applications, and the ability to penetrate skin [38,39,40,41].

SDF-1 (stromal cell-derived factor 1) plays pivotal roles in various physiological and developmental processes [42]. It primarily interacts with the CXCR4 receptor (C-X-C chemokine receptor 4) to facilitate cell migration, influence cell positioning within tissues, and contribute to the maintenance of tissue structure [43]. In addition, SDF-1 is specifically involved in stromal cell interactions and affects tissue architecture, neural development, and vascular formation [44]. In addition, it has a regulatory role in the immune response and is essential for embryonic development, particularly in the organogenesis of the cardiovascular and central nervous systems and B lymphopoiesis [44,45]. Furthermore, the interaction between SDF-1 and its receptor, CXCR4 (a G protein-coupled receptor), is crucial for regulating cell signaling, which, in turn, affects cell migration, differentiation, survival, and other vital functions [43]. CXCR4 is expressed by many cell types, including immune cells and those of the central nervous system and endothelium, which underlines the broad physiological impact of SDF-1 [46]. SDF-1 also plays key roles in anti-inflammatory processes by controlling the migration of immune cells, regulating cell survival and apoptosis, and reducing the expression of inflammatory mediators [47,48]. These functions underscore the importance of SDF-1 as a regulator in inflammatory conditions and highlight its involvement in the maintenance of tissue health and the protection it affords against inflammatory disorders [49,50].

In this study, we studied the anti-inflammatory effects of 18 small peptides derived from human SDF-1. Based on the ELISA results, we chose the most effective peptide, S10, which showed the greatest inhibitory effect on the production of IL-1β and TNF-α, the representative pro-inflammatory cytokines. S10 effectively inhibited the secretions of IL-1β and TNF-α in an in vitro inflammatory model of infection induced by *P. gingivalis* (Figure 1A) and demonstrates that S10 effectively suppresses the *P. gingivalis* infection-induced secretions of the pro-inflammatory cytokines IL-1β and TNF-α by THP-1 macrophages (Figure 2A). This observation suggests that S10 has potent anti-inflammatory properties and is a promising candidate for therapeutic intervention in inflammatory conditions. Also, S10 treatment attenuated the activation of NLRP3 and AIM2 inflammasomes, which were induced by *P. gingivalis* infection in this in vitro model, and this inhibitory effect extended to the expression of key participants in inflammasome assembly, such as ASC, caspase-1, and IL-1β (Figure 2B,C). Furthermore, S10 effectively reduced the formation of ASC specks, indicating an ability to disrupt the inflammasome assembly process (Figure 2D). This observation aligns with our previous findings [12], wherein we demonstrated that ASC specks serve as crucial platforms for the recruitment of pro-caspase-1, thereby facilitating the activation of inflammatory pathways. By inhibiting ASC speck formation, S10 interferes with this process, ultimately attenuating the downstream inflammatory responses triggered by *P. gingivalis* infection.

Mechanistic insights revealed that the anti-inflammatory effects of S10 are mediated through the suppression of the NF-κB and TLR2/4 signaling pathways. S10 downregulated the expression of TLR pathway components (Figure 3A) and inhibited the phosphorylation of NF-κB (Figure 3B) and MAPKs (Figure 3C), which are crucially required for the activation of inflammatory responses.

In addition, based on our previous study, we extended the study to an in vivo mouse model of experimental periodontitis [26,27]. In this study, we employed different experimental setups for the in vitro and in vivo experiments, to address the distinct requirements and objectives of each model. In the in vitro study, we administered S10 for 30 min before introducing *P. gingivalis* for 18 h, to assess the immediate cellular responses and the potential protective effects of S10 against the bacterial infection. This setup allowed us to control the environment closely and observe the direct impact of S10 on cells within a short time frame, providing insights into the initial interactions between S10, the cells, and the pathogen. Conversely, the in vivo study was designed to mimic a more realistic and prolonged infection scenario. The simultaneous administration of S10 and *P. gingivalis*, followed by a continuous 14-day treatment period, was intended to reflect a sustained therapeutic regimen that might be used in a clinical setting. This approach allowed us to evaluate the long-term effects of S10 on the progression of periodontitis in a living organism, considering factors like immune response, tissue interaction, and the overall health of the mouse model. The choice of administration times was based on previous studies and the specific goals of each experimental phase. For the in vitro study, a short pre-treatment period was sufficient to investigate the immediate effects of S10, while the extended treatment in the in vivo study was necessary to understand its potential as a long-term therapeutic agent. By using these different setups, we aimed to gather comprehensive data on the efficacy and mechanisms of S10 in both controlled and complex biological environments. We found that S10 administration significantly reduced the alveolar bone loss induced by *P. gingivalis* infection (Figure 4B,C), which highlighted the translational potential of S10 as a therapeutic agent for treating the inflammatory bone diseases associated with periodontitis. In this study, we also conducted observations on the gum’s toxicity, color, and condition. No changes were observed in these additional observations. However, conducting further toxicity and safety evaluations is necessary. Specifically, more experiments are needed to investigate the safety of S10 under various systems and conditions. Day 49 was chosen based on previous experiments. Namely, after those experiments, we determined that the time point of day 49 showed the best results under our current experimental conditions. This ability of S10 to modulate inflammatory responses and mitigate tissue damage underscores its therapeutic potential for the treatment of various inflammatory conditions, including periodontitis. By targeting multiple facets of the inflammatory cascade, including cytokine secretion, inflammasome activation, and signaling pathways, S10 offers a comprehensive means of attenuating inflammation and preserving tissue integrity. We surmise that S10 effectively regulates the inflammatory response because it contains specific active sites for SDF-1 and, like SDF-1, can induce signaling by binding to specific receptors, such as CXCR4 or CXCR7, and can thereby initiate signal transduction.

Further investigations are warranted to elucidate the precise molecular mechanisms underlying the anti-inflammatory effects of S10 and its potential off-target effects. In addition, studies are required on the safety, pharmacokinetics, and efficacy of S10 in larger animal models before clinical trials are conducted to validate its therapeutic utility in humans.

## 5. Conclusions

In summary, this study shows that S10 exerts an anti-inflammatory effect by inhibiting the activation of NLRP3, the AIM2 inflammasome, and the TLR2/4 signaling pathway. These findings demonstrate that S10 is a promising therapeutic agent that ameliorates the *P. gingivalis* infection-induced inflammatory response and offers new developmental routes toward targeted anti-inflammatory therapy.

## Data Availability

The original contributions presented in the study are included in the article material, further inquiries can be directed to the corresponding authors.
